# Farm Animal Cognition—Linking Behavior, Welfare and Ethics

**DOI:** 10.3389/fvets.2019.00024

**Published:** 2019-02-12

**Authors:** Christian Nawroth, Jan Langbein, Marjorie Coulon, Vivian Gabor, Susann Oesterwind, Judith Benz-Schwarzburg, Eberhard von Borell

**Affiliations:** ^1^Institute of Behavioural Physiology, Leibniz Institute for Farm Animal Biology (FBN), Dummerstorf, Germany; ^2^Centre for Proper Housing of Ruminants and Pigs, Federal Food Safety and Veterinary Office, Agroscope Tänikon, Bern, Switzerland; ^3^Cabinet EASIER, Clermont-Ferrand, France; ^4^Department of Animal Sciences, University of Goettingen, Goettingen, Germany; ^5^Faculty of Agricultural and Environmental Sciences, University of Rostock, Rostock, Germany; ^6^Unit for Ethics and Human-Animal Studies, Messerli Research Institute, Vetmeduni Vienna, University of Vienna, Medical University of Vienna, Vienna, Austria; ^7^Institute of Agricultural and Nutritional Sciences, Martin-Luther-University Halle-Wittenberg, Halle, Germany

**Keywords:** animal ethics, animal behavior, animal welfare, enrichment, human-animal interactions, livestock, physical cognition, social cognition

## Abstract

Farm animal welfare is a major concern for society and food production. To more accurately evaluate animal farming in general and to avoid exposing farm animals to poor welfare situations, it is necessary to understand not only their behavioral but also their cognitive needs and capacities. Thus, general knowledge of how farm animals perceive and interact with their environment is of major importance for a range of stakeholders, from citizens to politicians to cognitive ethologists to philosophers. This review aims to outline the current state of farm animal cognition research and focuses on ungulate livestock species, such as cattle, horses, pigs and small ruminants, and reflects upon a defined set of cognitive capacities (physical cognition: categorization, numerical ability, object permanence, reasoning, tool use; social cognition: individual discrimination and recognition, communication with humans, social learning, attribution of attention, prosociality, fairness). We identify a lack of information on certain aspects of physico-cognitive capacities in most farm animal species, such as numerosity discrimination and object permanence. This leads to further questions on how livestock comprehend their physical environment and understand causal relationships. Increasing our knowledge in this area will facilitate efforts to adjust husbandry systems and enrichment items to meet the needs and preferences of farm animals. Research in the socio-cognitive domain indicates that ungulate livestock possess sophisticated mental capacities, such as the discrimination between, and recognition of, conspecifics as well as human handlers using multiple modalities. Livestock also react to very subtle behavioral cues of conspecifics and humans. These socio-cognitive capacities can impact human-animal interactions during management practices and introduce ethical considerations on how to treat livestock in general. We emphasize the importance of gaining a better understanding of how livestock species interact with their physical and social environments, as this information can improve housing and management conditions and can be used to evaluate the use and treatment of animals during production.

## Introduction

Farm animal welfare assessment approaches have shifted from original concepts such as the five freedoms (1, 2) to more animal-centered concepts that also include the animals' needs (3), affective states (4, 5) and inter-individual differences (6). All these concepts emphasize the importance of having detailed knowledge on the cognitive capabilities of livestock [i.e., their ability to acquire, process, store and use information (7)] to better understand their behavior and to avoid exposing them to poor welfare conditions, such as those induced by stressful management practices. Thus, cognitive research on farm animals has the potential to highlight mismatches between current husbandry practices and adaptive abilities of livestock.

In recent decades, research on the cognitive capacities of non-human animals has gained increasing attention. Most work has focused on humans' closest relatives, i.e., primates in general and great apes in particular (8); additionally, in terms of convergent cognitive evolution, research has included corvids (9) and canids (10). However, compared to the amount of cognitive research that has been conducted on the aforementioned species, studies on the cognitive capabilities of farm animals are relatively underrepresented (11). Given the number of livestock animals kept under husbandry conditions worldwide, this lack of research is even more surprising.

The aim of this review article is to highlight advances in the field of farm animal cognition and identify their potential implications for livestock management and practices. First, we outline a distinct set of physico- and socio-cognitive processes. We then present a comprehensive overview on farm animal cognition research conducted on cattle, horses, pigs, and small ruminants. Our purpose is to offer a structural account of the content and value of these processes that can help researchers further explore the different cognitive mechanisms in a variety of farm animals to gain a more comprehensive profile of species-specific psychological traits. We then discuss the implications of these findings on issues concerning animal welfare (while focusing on enrichment and human-animal interactions) and ethics (comprising welfare ethics and ethical considerations beyond animal welfare). Finally, we offer directions for future research that will help to further our understanding of farm animal cognition and help us to adapt management practices that better meet their psychological needs.

We structured this review mainly in line with the classification of cognitive capacities used by Shettleworth (7). She broadly categorized cognitive mechanisms into two domains: physical and social cognition. We focused on a particular set of cognitive capacities in both domains, which we assume to have a major impact on how farm animals are able to interact with their physical and social environment (see Tables 1, 2 for an overview).

**Table 1:** Overview on various physico-cognitive capacities, their description, evidence in different farm animals, and their implications.

**Cognitive trait**	**Description**	**Cattle**	**Horses**	**Pigs**	**Small ruminants**	**Implications**
Categorization	Ability to group items based on common features	+ (12)	+ (13, 14)	n/a	+ (15) (16)	Adaptation to novel stressors (food acquisition, handling)
Numerical ability	Discrimination and judgment of distinct quantities	n/a	+ (17, 18)	n/a	n/a	Perceived predictability of environment (group number) and adaptation to stressors (group cohesion)
Object permanence	Notion that objects continue to exist when they move out of the visual field	n/a	(+) (19, 20)	± (21)(+) (22)	+ (23)	Perceived predictability of environment (housing)
Reasoning/Inferences	Establishment of an association between a visible and an imagined event	n/a	n/a	+ (24)	+ (goats) (25) – (sheep) (25)	Perceived predictability of environment (housing); Complexity of cognitive enrichment
Tool use	Manipulation of objects to reach a goal	n/a	n/a	n/a	n/a	Complexity of cognitive enrichment

*+, positive results; (+), indirect positive results; ±, inconclusive results; –, negative results; n/a, no studies available*.

**Table 2:** Overview on various socio-cognitive capacities, their description, evidence in different farm animals, and their implications.

**Cognitive trait**	**Description**	**Cattle**	**Horses**	**Pigs**	**Small ruminants**	**Implication**
Discrimination and recognition of conspecifics	Differentiating and recalling other individuals	+ (12, 26)	+ (27, 28)	+ (29, 30)	+ (31, 32)	Group cohesionReduction of aggressive behavior
Discrimination and recognition of humans	Differentiating and recalling handlers	+ (33)	+ (34, 35)	+ (36, 37)	+ (38)	Stockmanship (fear response to familiar/unfamiliar humans)
Communication with humans(Human → Animal)	Use of human communicative cues, such as a pointing gesture	n/a	+ (19, 39)	+ (22, 40)	+ (41, 42)	Management and stockmanship during handling and transport
Communication with humans(Animal → Human)	Expression of communicative behaviors, such as gaze alternations between a human and an object	n/a	+ (43)	n/a	+ (44, 45)	Signaling of needs
Social learning (vertical)	Information transfer from parents to offspring	± (46)	+ (47)	+ (48, 49)	+ (50)	Access to resources and avoidance of harm
Social learning (horizontal)	Information transfer from peer to peer	± (51, 52)	± (53, 54)	+ (55)	– (56, 57)	Group organization and access to resources
Social learning (from humans)	Information transfer from humans	n/a	± (58, 59)	n/a	+ (60)	Adaption to new environments
Attributing attention	Attending to signs of attention in conspecifics or humans (i.e., head direction or eye visibility)	n/a	+ (61)	± (62–65)	+ (42, 66)	Predictability of events/actions/ interactions; perceived access to resources
Prosocial behavior	Behavior that benefits other individuals and their welfare	n/a	n/a	n/a	n/a	Ethical implications
Fairness (inequity aversion, third party punishment)	Behavior regarding the outcome of decision as equal and just toward oneself and others	n/a	n/a	n/a	n/a	Ethical implications

*+ positive results; (+), indirect positive results; ±, inconclusive results; –, negative results; n/a, no studies available*.

### Physical Cognition

The term “physical cognition” refers to an organism's understanding of objects and their various spatial and causal relationships. For most animal species, the most important problem they face is related to locating and obtaining food. Thus, many important cognitive skills have evolved in the context of foraging (8).

*Categorization* is the ability to group items based on common features and to respond similarly to them. This trait enables an organism to group together objects and events based on physical, associative or relational similarities, and it provides the basis for higher cognitive processing (67). In complex environments, the ability to assign food types to categories by relying on certain relevant criteria could considerably reduce cognitive demand and thereby increase foraging efficiency. Being able to categorize positive and negative stimuli might also enhance adaptations to new environments, which could reduce the impact of stressors such as food acquisition in novel environments or handling during transport.

*Numerical ability* refers to the capacity to discriminate between two distinct quantities (e.g., 6 vs. 4 rewards or 3 vs. 1 conspecifics), regardless of the size and shape of these objects/subjects (68). Several mechanisms, such as “subitizing” or “approximate number system,” can explain this phenomenon (69). The ability to assess the quantity of food or members of a group likely affects the predictability of the environment (e.g., group number) and the ability to adapt to stressors (e.g., group cohesion).

*Object permanence* refers to the notion that objects are perceived as separate entities that continue to exist even when they are out of the sight of the observer (70). Following the trajectory of a previously seen but then hidden object is highly adaptive in the context of foraging and also helps to avoid predation. As husbandry systems involve a variety of barriers and walls, the degree of how well object permanence is developed plays a crucial role in being able to predict upcoming events in farming environments.

*Reasoning (inferences)* implies the establishment of an association between a visible and an imagined event (71). The correct solution to a problem should be selected by excluding other potential alternatives even if only indirect information, such as the absence of a cue, is available. However, inferential reasoning can only be assumed if the subject exhibits adequate behavior, without explicit training. Otherwise, the role of learning mechanisms cannot be excluded (72, 73). The ability to reason about events likely impacts the predictability of husbandry environments.

*Tool use*, i.e., the ability to dynamically manipulate and to use an inanimate object (or animate subject) to reach a goal, is a topic that has gained interest in non-human cognitive research in recent decades and has been found in several animal taxa (74, 75). Goals can be manifold, i.e., the acquisition and gathering of food and water, grooming, self-defense, or recreational use. Animals that regularly use tools can be provided with a higher diversity (and complexity) of enrichment items (e.g., arbitrary anthills for chimpanzees).

### Social Cognition

Conspecifics are physical objects that must be located and identified, but they also create additional cognitive problems that are not present in the world of inanimate objects (8). For example, living in groups requires the discrimination and recall of conspecifics, either at the individual or group level. Being a social animal also may require additional forms of intelligence, such as manipulating the behavior of others (76). Moreover, another individual might behave spontaneously on its own. Thus, the ability to infer the motivations and desires of others can be advantageous in terms of lowering the level of uncertainty in predicting the behavior of others.

*Individual discrimination and recognition* of conspecifics and heterospecifics are considered complex processes that have evolved to facilitate social behaviors. Discrimination refers to the ability to differentiate between two identities (e.g., individual conspecifics or heterospecifics, such as humans) using cues inherent to these individuals. Individual recognition requires the ability to remember and recall other individuals (77). An essential feature of individual recognition in humans is that it is cross-modal, which means the trait enables the matching of current sensory cues of identity with stored information about that specific individual from other modalities (78). Being able to recognize conspecifics after short- or long-term periods of separation reduces aggressive behavior and injuries, while being able to correctly identify familiar handlers likely reduces stress during management practices.

*Communication with humans* can be crucial for domestic animals in terms of acquiring information from the environment (79). Several non-human animals have been shown to use human-given cues, such as a pointing gesture, when confronted with a task where they have to make a choice between two or more potential alternatives of where to find hidden food. In turn, communication can also be directed toward humans. When confronted with a problem they cannot solve themselves, children as well as some non-human animals use gazing behaviors (such as i.e., alternating their gaze between the problem and a human) as a form of referential and intentional communication when interacting with humans (80–82). Thus, the ability of livestock to communicate with humans can impact on management practices due to improvements in handling routines.

Another crucial part of social cognition involves *social facilitation and/or learning* [for a distinction see (83), from here on referred to as “social learning”]. These processes can occur through observational conditioning, local/stimulus enhancement, emulation and/or imitation (84). Social learning occurs when a subject's behavior is influenced by observing the behavior of other individuals, and it often arises when individual learning is costly, e.g., in terms of predation risk or offspring foraging behavior (85, 86), and is not limited by mechanistic constraints (87). Social learning can occur horizontally (i.e., from peer to peer) or vertically (i.e., from parents to offspring, but also from unrelated adults to young in general). It is apparent that acquiring new information in husbandry systems (e.g., where to find food) through social learning has huge advantages, e.g., reduced stress and increased food intake.

An individual's knowledge of the perceptual states of others, which is comprised by the ability to *attribute attention* or take the perspective of another individual, can be summarized under the so-called “Theory of Mind” or “Theory of Mind”-like abilities (88). Attributing attention to conspecifics or handlers can increase the predictability of future interactions and events (conspecifics: competition for access to resources; handlers: management practices).

Finally, the domain of social cognition also involves questions regarding the cognitive foundations of morality, e.g., actions involving the welfare of others, such as *prosociality* (89) and *fairness* [including inequity aversion and third-party punishment (90)]. The capacity for prosocial behavior can be used to promote positive mental states and well-being in social farm animals (91) while it also raises ethical questions regarding the use of animals in general.

## Cognitive Studies in Livestock Animals

### Cattle

#### Physical Cognition

Previous research in cattle has primarily focused on their learning ability rather than on their understanding of physical properties of their environment (92, 93). Cattle associate locations with the quantity and quality of food that is found there (92), and they adjust their foraging patterns to take advantage of this knowledge, which indicates context-dependent decision making. They also appear to have a categorization process for social stimuli: Cattle categorize individuals into “familiar” and “unfamiliar” subjects (12). However, no studies regarding number discrimination, object permanence, reasoning or tool use are available for this species.

#### Social Cognition

Cattle have social recognition abilities, which include individual recognition (77). There is also indirect evidence of discrimination based on familiarity, which is one of the simpler categories of social recognition. For example, intense fighting between cattle frequently occurs when groups of unfamiliar individuals are mixed at abattoirs, e.g., heifers were less aggressive to familiar members than to unfamiliar animals (94). In their herd, subordinate cattle will generally avoid more dominant animals, which suggests they have the ability to recognize familiar animals that have been previously associated with positive or negative experiences (95). Individuals within a herd also prefer the presence of social partners with whom they have already maintained close proximity and direct social grooming (94). Similarly, dam and offspring form strong social bonds and are able to recognize each other even within a large herd (96). Categorizing individuals based on familiarity, social status and genetic relatedness is important for social cohesion in cattle; moreover, these skills decrease aggression within a group and help categorize individuals as kin and non-kin.

Operant conditioning techniques have been used to test cattle in terms of their ability to discriminate and categorize individuals. Cattle easily discriminate among familiar conspecifics using visual, olfactory and auditory modalities (97), and they can be trained to discriminate between conspecifics using only olfactory cues (98). Cattle appear to use their sense of vision to discriminate between conspecifics, as altering their vision ability resulted in an increase in the frequency of aggressive interactions (99). Coulon et al. (12) tested the ability of cattle to visually discriminate between heads (including face views) of familiar and unfamiliar conspecifics represented as 2D images using a food-rewarded instrumental conditioning procedure. Eight out of the nine heifers succeeded in discriminating between images of familiar and unfamiliar conspecifics; furthermore, they could instantly differentiate between a new pair of images of familiar and unfamiliar conspecifics, suggesting cattle have a categorization process for social stimuli. In addition, Coulon et al. (12) argued that images of conspecifics were treated as representations of real individuals. Indeed, they observed that heifers were more attracted to images of familiar conspecifics than to images of unfamiliar conspecifics. In addition, heifers expressed different emotional reactions when confronted with these two types of stimuli. Heifers rewarded for images of unfamiliar conspecifics pointed their ears backwards more frequently (which is a behavior common during the confrontation with an unfamiliar and potentially threatening stimuli), and they showed less forward pointing ears (indicating less positive expectations) when they directed their attention to unfamiliar images compared to heifers that were rewarded for identifying familiar conspecifics (100). Using the same methodology, heifers visually discriminated their own species from other animal species (26) and kin-related conspecifics from non-kin conspecifics (101). It appears that the heads of conspecifics are sufficient for social discrimination in cattle. Several authors have shown that cattle can discriminate between humans using various criteria (102), such as a portion of their face or their body (33) or the color of the clothes of unfamiliar people (103).

There is evidence of social facilitation/learning in cattle. For example, calves can learn where to graze from their dam (46). If a cow is located in an area with high food resources, this information is transferred to other members of the social group by social facilitation (51). In addition, conditioned aversions have been eliminated following exposure to non-averted social companions in cattle (104). Although heifers provided with a trained demonstrator did not learn an operant task faster, they spent more time near the training box (52). Boissy and Le Neindre (105) also found that the degree of familiarity between heifers did not affect their social learning abilities in relation to an operant task.

To the best of our knowledge, there have been no studies on social learning from humans or the use of human-given cues in cattle. In addition, no studies have focused on more complex socio-cognitive phenomena, such as prosocial behavior or inequity aversion, in cattle.

### Horses

#### Physical Cognition

Horses can learn to choose items in a choice task based on the shared characteristics of these items (e.g., filled item vs. items with an opening), indicating that they are using categorization skills when confronted with problem-solving tasks (13, 14).

Several investigations have evaluated the ability of horses to discriminate between different quantities. For example, Uller and Lewis (18) showed a spontaneous preference of horses to choose the higher quantity of plastic apples regardless of the total volume. In this study, the horses spontaneously chose the higher quantity in the 1 vs. 2 and in the 2 vs. 3 tasks, but they failed to discriminate between 4 vs. 6 apples. In the study by Henselek et al. (106), horses failed to discriminate pairs that differed in the number of edible and inedible objects (i.e., apple slices and small wooden blocks). On the other hand, Petrazzini (107) found that a horse could discriminate pairs of dots when they were presented under uncontrolled conditions (i.e., the ratio of surface was similar to the ratio of number) and controlled conditions (i.e., equal surface area over whole stimulus) in a 1 vs. 4 task, but the horses failed in the higher ratio of 2 vs. 4 in the controlled condition. She assumed that if discrimination becomes more difficult, horses also tend to use cues other than numerical cues. In each of the mentioned experiments, the positive stimulus was the one with the higher quantity of items. The fact that the horses in these studies failed to discriminate higher quantities or higher ratios could lead to the presumption that the animals based their decisions on approximate estimations. Gabor and Gerken (17) found that horses are able to discriminate the quantity of abstract symbols. Three horses could discriminate various ratios (1 vs. 2; 2 vs. 3; 3 vs. 4; 4 vs. 5), and one horse was able to transfer the performance to mixed geometrical symbols.

No research has directly investigated the ability of horses to track hidden objects (i.e., object permanence); however, results from experiments that have used hiding containers (18) have indirectly provided evidence for at least rudimentary developed object permanence skills. No studies regarding reasoning or tool use are available for this species.

#### Social Cognition

Horses are able to differentiate between conspecifics (27, 108) and humans using different modalities (34), and can identify subjects based on familiarity (109, 110). Previous research has shown that horses are also able to recognize conspecific and heterospecific individuals across two modalities (i.e., the individual visual and auditory cues must match) (28, 35, 111).

Horses are known to react to subtle behavioral cues from conspecifics and humans (112). Surprisingly, the general body posture used when approaching a horse did not appear to have an influence. However, the speed of the human's approach was more influential (113). In contrast to these findings, Proops and McComb (61) and Krueger et al. (20) found that horses differed in their approach behavior to humans based on the level of attention that was provided by an experimenter. The horses also showed higher obedience levels when a human was giving them attention (110). Several studies have reported that horses are able to use human-given cues, such as a pointing gesture, to locate a hidden reward. However, it seems that the pointing finger must be close to the baited location, as performance dropped toward levels equal to random chance when pointing was administered from a distance (19, 39, 114–116). In contrast to the use of human pointing gestures, horses failed to interpret the head direction of a human experimenter (19, 116), but they were able to use the head direction of a depiction of a conspecific to infer the location of a hidden reward (117). Horses appear to wait for humans to solve a task to obtain food instead of trying to solve the task themselves (118). They also show human-directed behavior when confronted with an inaccessible food reward (43), and they frequently gazed at an experimenter who was positioned near the reward. In addition, horses also considered the attentional stance of the experimenter during the task, depending on whether the experimenter was turned toward or away from them.

Horse owners often think that abnormal or stereotypic behaviors are learned through observation (119); however, several studies have shown that horses do not perform better after watching a demonstrator horse both in a simple operant conditioning task (120) or in a discrimination task (121–123). Krueger and Heinze (124) found that experimental horses copied specific following behaviors toward humans when a dominant conspecific followed the path of a human handler. However, studies in horses on imitation of complex behaviors have shown inconclusive results so far, which may be due to the lack of appropriate experimental designs (125). Recently, Ahrendt et al. (126), Rørvang et al. (54), and Burla et al. (58) showed that horses do not learn an instrumental or spatial task through social observation. In contrast, a study by Krueger et al. (53) found evidence for social learning in horses using an instrumental task. However, this was restricted to young, low-ranking and more exploratory horses who were learning from older group members. Additionally, test horses learned the same instrumental task faster than control horses when they were frequently exposed to a human demonstrator who was solving the instrumental task (59). When mares were habituated to the exposure of a human experimenter or unfamiliar and potential frightening objects, their foals showed less fear reactions in standardized fear tests or in approaching unfamiliar humans, indicating the social transfer of information from mother to offspring (47, 127).

No studies on the evaluation of more complex socio-cognitive phenomena, such as prosocial behavior or inequity aversion, are available.

### Pigs

#### Physical Cognition

Pigs as omnivorous animals exhibit high foraging flexibility that is reflected in their dietary spectrum (128). Therefore, it should be of no surprise to find cognitive capacities that increase their ability to exploit food sources, either in relocating previously known food patches or in finding new ones.

Young pigs have been found to understand that once hidden, objects do not cease to exist, but had problems following more complex movements of hidden objects (21). Albiach-Serrano et al. (40) presented domestic pigs with a series of tasks that spanned the physico- and socio-cognitive domains. One of these tasks involved the presentation of a slighted board that covered a hidden reward, and another task involved a baited cup that was shaken to produce a rattling noise. Subjects had to infer the position of the reward by interpreting the causal relationships between the reward and the board/cup, i.e., the inclination of the board or the noise that was generated by shaking the cup. Although pigs could solve the tasks, it was unclear whether they simply relied on stimulus enhancement cues (i.e., the slope of the board and the shaking movement or noise of the cup). Nawroth and von Borell (24) repeated the latter task that used a shaking bucket with a modified setup. Here, pigs were tested in their ability to use indirect visual and auditory stimuli (i.e., the absence of visual or acoustic cues) by choosing between two potential hiding locations. Pigs used indirect visual cues and, to some degree, indirect auditory cues, i.e., the absence of food by lifting one bowl or the absence of noise during the shaking of the bowl, to infer the location of the hidden reward. Again, the experimental design could not exclude the possibility that pigs were simply avoiding the non-rewarded location and relied on learned contingencies. However, these results provide support that pigs can rapidly adapt to new foraging situations.

Although it was demonstrated that pigs were able to differentiate between different amounts of food (129, 130), more complex studies that evaluated numerical competence should be conducted to investigate the cognitive mechanisms involved in this process. In addition, no studies involving categorization abilities or tool use have been conducted with pigs.

#### Social Cognition

Pigs are highly gregarious animals and thus establish stable social hierarchies. This requires good discriminatory abilities to differentiate between group members and between familiar and non-familiar individuals. Studies found pigs were able to distinguish unfamiliar from familiar conspecifics (131); additionally, pigs could differentiate familiar individuals using visual, auditory or olfactory cues alone (29, 30). However, 2D head cues were insufficient for pigs to discriminate between familiar conspecifics (132); thus, features other than head cues may be more salient for pigs. For example, studies on the ability of pigs to visually discriminate between humans showed that pigs mainly relied on the body height and upper torso of the human (36, 37, 133).

Nawroth et al. (65) used an approach that focused exclusively on the differentiation among the attentive states of humans. Juvenile pigs had to choose between two unfamiliar persons, while only one human focused attention on the test subject; the test conditions varied, and it was assumed that only the attentive human would provide food immediately or would provide food at all. While the subject performance in the choice task was poor and the results were inconclusive, two approach styles were distinguished during decision making. Here, pigs chose the attentive person more often when they approached non-impulsively (i.e., changed direction of or paused during approach), which was not the case when subjects chose impulsively (i.e., went straight to one person).

An object choice task is another well-known test used to investigate heterospecific communicative abilities. Here, subjects must choose between two potential baiting locations, of which only one contains a reward. To find the reward, a human administers different types of cues (e.g., pointing or gazing) toward the baited location (134). Given that pigs, unlike dogs, are not domesticated for companionship but are commonly raised as meat stock, their human social environment is often less demanding than that of dogs, and this might hamper their inclination to rely on human-given cues. This was partly supported by the findings of Albiach-Serrano et al. (40), who reported inconclusive results in the use of human-given cues (e.g., pointing, head orientation) by pigs. In contrast, Nawroth et al. (22) provided evidence that even very young pigs were able to use a variety of pointing cues and were also able to utilize the body and head orientation of a human experimenter to locate a hidden reward. However, pigs could have learned the gestures rapidly or, in terms of the pointing gestures, relied on stimulus enhancement. Thus, it is not clear whether the pigs were able to comprehend the referential and intentional nature of the human-given cues or whether they used learned contingencies to solve the task [see (134)].

Only a few studies have shown that pigs seem to be capable of social learning, either vertically (48, 49) or horizontally (55). However, in most examples, learning was directly related to food cues and could have been acquired through direct snout-snout interactions rather than visual observation. Based on their foraging ecology, it would be advantageous to not only learn what to eat but also learn how to acquire und process particular food sources. Recently, it has been demonstrated that juvenile Kunekune pigs learned how to manipulate objects (i.e., open a door) to receive a reward from related adult individuals (49). However, there have not been any studies on horizontal social learning that involves problem-solving or object manipulation for pigs.

Pigs are highly competitive foragers, and they rely on patchily distributed food sources. Therefore, it is unsurprising that dominant pigs readily start to scrounge on subordinate individuals of the group by following them to food patches they have discovered (135). In terms of this exploitation, it seems adaptive to be aware of the presence and attentive states of other individuals. Indeed, research suggests that pigs are able to attribute attentive states toward other individuals. Using an informed forager paradigm (136), Held et al. (63) found that the approach time to a baited container of a subordinate but knowledgeable pig depends on the body position of a dominant but ignorant conspecific. Overall, the subordinates were more likely to show food-directed behavior when the chances of arriving at the food source ahead of their exploiters were higher. Intriguingly, subordinates adjusted their foraging behavior based on whom they were foraging with, i.e., counter-exploitation behavior was only observed with dominant subjects that had already scrounged on the subordinates in previous foraging trials (137). In another study, Held et al. (62) allowed pigs to follow two companion pigs, of which one was able to see the baiting of food and the other was not. Most pigs did not follow their companions, likely to avoid competitive and aggressive behavior. Nonetheless, out of ten pigs, two subjects followed their conspecifics, and one of them followed the “knowing” individual significantly more often than the “unknowing” individual. These studies suggest that pigs use body cues to discriminate between the different attentive states of conspecifics and that they, to some degree, might be able to interpret the visual perspective of others. No studies have focused on more complex socio-cognitive phenomena, such as prosocial behavior or inequity aversion, in pigs.

### Small Ruminants

#### Physical Cognition

Goats and sheep are both small ruminants that preferably graze/browse on grass and herbs; thus, being able to categorize food stimuli should be useful to these animals during foraging. For example, sheep have been shown to use species-based categorization when selecting their diet. Sheep generalized their aversion among species and classes of plants into distinct categories (15, 138). Experiments under husbandry conditions have also shown that goats have more abstract learning and categorization abilities. By using an automated learning device, Langbein et al. (139) investigated “learning to learn” or “learning set” formation in dwarf goats. The performance of the animals improved when the animals were tested using a series of visual discrimination tasks, and the results indicated that the goats started to develop a “learning set.” In addition, dwarf goats can form open-ended categories based on similarities in the visual appearance of artificial symbols, while individuals are also able to generalize these categories across new symbols (16). Using a similar experimental setup, individual goats were found to be capable of learning the oddity concept. When presented with an odd stimulus and three identical non-odd stimuli on the automated learning device, these animals consistently chose the odd stimulus after the initial training (140).

Small ruminants possess a sophisticated understanding of their physical environment. When confronted with a task in which a reward was hidden in one of two opaque containers that then switched positions, the goats showed moderate to high success rates in finding the reward (23). Individual goats, but not sheep, can also infer the location of a reward through exclusion. When provided with a choice between two containers (while only one was baited with a reward), goats and sheep were able to use direct information (i.e., the presence of food) from the baited container in the choice task. However, only goats, but not sheep, used indirect information (i.e., the absence of the reward) from the empty container to infer the presence of the reward in the baited container (25). Due to the different feeding preferences of goats (low-fiber feeders; dietary browsers) compared to sheep (high-fiber feeders; dietary grazers), goats might prefer and forage more selectively than do sheep. This higher flexibility may have led to the avoidance of a potential, but empty, food location in goats but not in sheep. In fact, an earlier study by Hosoi et al. (141) indicated that goats avoided high-fiber food when they were offered the option to feed on low-fiber food, but sheep did not. No studies have investigated number discrimination or tool use for either goats or sheep.

#### Social Cognition

Goats and sheep live in fission-fusion societies with stable dominance hierarchies (142, 143); thus, it should be highly advantageous for them to remember and recognize familiar group members. When presented with pairs of face images or vocalizations, sheep were able to discriminate between different species (including humans), breeds and sexes of their same breed. The sheep learned to distinguish between individual adult sheep faces, but breed and social familiarity influenced the level of discrimination performance (144, 145). Sheep behavioral and neural activities also indicated they remembered faces of familiar conspecifics after more than 2 years, which suggests sheep have a high capacity for learning and memory (31); moreover, that 2D images of conspecific faces seemed to be represented as a 3D equivalent of the real-life individual. Additional evidence for this was reported in a recent experiment where sheep recognized a familiar handler when the face of this handler was presented as a 2D image in a discrimination task (38).

Goats can differentiate among conspecifics using visual and/or acoustic cues (32, 146, 147). For example, Keil et al. (32) showed that goats discriminated between familiar and unfamiliar conspecifics, even when their heads were not visible. Surprisingly, there have been no investigations to determine how goats discriminate between humans.

There is broad popular interest in the relationship between humans and small ruminants, specifically on how small ruminants react to being observed by humans (e.g., the movie “The Men Who Stare at Goats”). Indeed, human gaze appeared to alter the behavior of domestic sheep compared to situations where there was no human eye contact (148): Sheep glanced at the gazing human more often and showed higher levels of activity. Nawroth et al. (42, 149) found that goats differed in their anticipatory behavior depending on a human's attentive state. For instance, when an inaccessible reward was positioned in front of a goat, an experimenter engaged in different postures that resembled different levels of attention toward the subject (e.g., back turned toward the subject or eyes closed). The anticipatory behavior of goats increased as the experimenter gave more attention to the subject, while alert behavior (“standing alert”) was most prominent when the experimenter was present but not giving the test subject any attention. These results indicated that the goats adapted their behavior based on the head and body orientation, but not the eye visibility, of the experimenter as a means of being given a reward. The results related to the body orientation were confirmed in a different experiment that used a choice paradigm; specifically, goats could choose to beg for food from either an attentive or inattentive person (i.e., body turned toward subject vs. body turned away). However, the head orientation of humans did not affect the choice behavior of goats (66).

Nawroth et al. (42) and Kaminski et al. (41) investigated the ability of goats to use various human-given cues in an object-choice task to locate a hidden reward. The authors found that goats were able to utilize human pointing gestures, but goats could not interpret the head or gaze direction of a human to find the hidden reward. In contrast to the negative findings regarding the human head and gaze direction, goats could follow the gaze direction of conspecifics into distant space (41), which is an extremely important trait in terms of predator detection (150). Goats also showed human-directed behavior in the form of frequent gaze alternations toward humans when they were confronted with an inaccessible food reward (44, 45), which was similar to what has been found in dogs and horses (43, 81). Here, as well, goats considered the attentional stance of the experimenter and altered their use of gaze alternations during the task depending on whether the experimenter was turned toward or away from them.

In small ruminants, vertical information transfer between individuals (e.g., social learning by offspring from adults) is important for the development of foraging skills (50, 151). For example, lambs can learn how to use an artificial teat from knowledgeable lambs that where transferred into their group (152). Baciadonna et al. (56) tested goats in a foraging task where they had the opportunity to follow another goat in a Y-maze or to rely on their own experience where to find a reward. Goats relied more on personal information than on social information when both types were available and conflicted with each other. Briefer et al. (57) investigated social problem-solving abilities of goats using a complex two-step foraging task in which subjects had to first pull a rope and then lift a lever to receive access to food. Goats quickly learned the task on an individual basis. However, goats that observed a demonstrator goat first did not learn the task faster compared to goats that did not see a demonstration. This indicated that the goats relied on individual experience rather than on social experience in this particular task. In contrast to these previous findings, human demonstration improved goats' performance in a spatial problem-solving task (60). Goats that experienced a human demonstrator detouring a V-shaped hurdle solved the detour faster compared to goats that did not receive a demonstration. No studies are available regarding more complex socio-cognitive phenomena, such as prosocial behavior or inequity aversion.

## Impact on Welfare Practices

General knowledge of how farm animals perceive and address their physical and social environment is of interest for improving housing and management practices; it can also be used in future studies in the different fields of applied ethology (64, 153–155).

### Enrichment

Livestock housing conditions are often structurally simple and offer limited possibilities to exhibit species-appropriate behavior (156, 157). These limitations can lead to boredom and frustration, which promotes the appearance of abnormal behavior, especially that which is related to stress and reduced welfare (158, 159). One way to decrease the level of boredom and frustration in livestock is to enhance the biological relevance of the housing conditions of farm animals; this can be done through the provision of a variety of new structures, items and challenges that are related to the animals' needs and natural behavioral repertoire (157). This so-called environmental enrichment is supposed to elicit a higher degree of behavioral diversity by increasing the physical and social complexity of the livestock environment (160, 161). Providing specific cognitive enrichment, e.g., artificial challenges associated with rewards, should, through positive reinforcement and the associated control and predictability of the environment, evoke positive affective states in livestock and improve their wellbeing (162–164). A detailed understanding of the cognitive capacities of farm animals, and especially their understanding of the physical properties of their environment, will provide help to design proper forms of structural and cognitive enrichment (165–168).

The integration of different types of cognitive enrichment into the housing of farm animals has received little attention; to date, approaches have been based on the instrumental or operant learning skills of subjects. For example, using a computerized feeding device (“call-feeding station,” CFS), piglets were required to recognize an individual sound signal and then to operate a button at an increasing fixed ratio to receive a food reward (169). Animals learned the task, which was taught using a combination of classical and operant conditioning, within a short time period. After several weeks of receiving food via the CFS, piglets showed less stress during feeding and evoked longer lasting positive emotions (170) compared to the control animals; moreover, the piglets displayed less abnormal behavior and showed reduced signs of fear in the context of being faced with a challenging environment (169). When goats were successively confronted with several different visual discrimination tasks through the use of a computer-based learning device that was integrated into the home pen, their heart rates initially increased but then decreased as the goats showed increased learning performance in consecutive tasks (171). This indicated that the goats had been exposed to a challenging task that induced positive eustress (172). It also appeared that goats seemed to seek challenges; for example, goats continued to operate the rewarding learning device even when an identical reward was available without the requirement of additional cognitive effort (173). This behavioral pattern is linked to the concept of contrafreeloading (174) and indicates that successfully coping with a cognitively challenging device or procedure could have intrinsic reinforcing properties beyond the extrinsic reward itself. Further evidence of this has been provided in experiments on heifers and beagles, who showed greater positive excitement after learning an operant task than did control animals who did not have to solve the task themselves. This excitement that accompanies success is believed to be related to positive affective states in non- human animals (175, 176).

Regardless of which device or procedure is used to cognitively challenge the animals, the device or procedure must be modified regularly to be remain challenging, e.g., by changing the rewarded cue or the entire task (177). Otherwise, the animals will develop routine-like behavior, and the device will no longer be challenging and enriching. On the other hand, to develop appropriate challenges that do not overstrain the animals, we need to have detailed knowledge on the species-specific problem-solving abilities of livestock animals.

As described, the first attempts to integrate cognitively challenging tasks into housing to promote cognitive enrichment have focused on operant conditioning tasks. In the future, in addition to relying on learning, it may be important to also rely on physico-cognitive traits (such as categorization abilities or making inferences) to pave the way for new opportunities on how to integrate changing challenges into the housing environments of animals (Table 1). However, the limited availability of solid evidence of the physico-cognitive capacities discussed in this review demonstrates how little we actually know about the problem-solving abilities of farm animals and their perception of their physical environment.

### Transfer

When farm animals are transferred to new environments during ontogenesis and confronted with new devices, e.g., automatically delivered food or water or offered comfort, they often need time to acclimate to these new conditions and learn how to use the devices (178). During these situations, it might be highly beneficial to rely on the mechanisms related to social or observational learning from experienced conspecifics (or humans) who act as demonstrators; this may facilitate the adaptation process to the novel housing conditions. To achieve this, we must identify the distinct and species-specific mechanisms of social learning in farm animals (see Table 2). In sheep, social learning plays an important role in the transmission of diet preferences (179). Housing dairy calves in social groups results in increased weaning weights compared with calves that have been individually housed; this result is likely due to the increased intake of dry matter, which is often attributed to social learning or social facilitation during feeding (180, 181). In lambs, learning to suckle from an artificial teat was facilitated when an experienced partner was in the group compared to the control group that did not have a demonstrator. Experimental lambs sniffed or sucked the teat more often than did the lambs in the control group (152). Future research should identify which potential mechanisms, e.g., social facilitation, stimulus and local enhancement, or observational conditioning (86, 182), enable farm animals to use information from conspecifics or heterospecifics.

### Human—Animal Interactions

To improve handling practices under farm management conditions, it is important to know how livestock perceive and interact with humans. Based on this knowledge, applied research can be better adjusted to assess how subtle human behavioral changes can have rewarding or adverse effects on livestock behavior (183). During recent decades, it has been shown that different farm animal species can discriminate between individual humans (see Table 2), and animals may use individual humans to predict positive or negative events that are routinely involved in housing and management (184, 185). Mini pigs that were positively reinforced by their handlers over several weeks discriminated between the familiar keeper and a stranger in a Y-maze test using auditory, visual and olfactory cues (133). Similarly, cattle have been shown to discriminate between a handler who reinforced an operant action and a handler who did not (102). A differential reaction towards humans has been observed in sheep; for example, lambs handled by an unfriendly handler generalized their fear responses toward familiar and unfamiliar humans, while gently treated lambs discriminated between familiar and unfamiliar humans (186). Horses have also been shown to generalize their experiences with positive and negative stockpersons from one human to another (187). This gradual variation in the abilities of different livestock species to differentiate between individual humans based on their attitude toward the animals might have profound implications for animal housing and management.

Next, to avoid negative impacts, it is also important to identify and implement rewarding human-animal interactions (183). Studies on tactile human-animal interactions have demonstrated that there is potential to identify relevant stress-reducing behavior by stockpersons during handling and transport processes (188–191). For example, direct interactions between farm animals and their handlers (e.g., gentle touching or stroking) resulted in the animals having reduced stress and fear of humans (192–194); therefore, this improved the ease of handling, productivity and immune response (195–197).

Furthermore, various farm animal species follow human-given communicative cues and differ in their behavior based on whether a human gives them attention or not (see Table 2). Although most of these experiments included (previous) positive feedback from humans, animals will also likely show different responses based on the attentional stance of a human in more aversive settings, e.g., routine handling practices (148). Some livestock species, such as goats and horses (43, 44, 118), have been shown to also engage in communication efforts directed at humans (Table 2). Animals used behaviors, such as gaze alternations, to direct the attention of a human toward a problem that the animal could not solve themselves. In terms of applying this to the farm, the skilful reading of these cues could lead to the improved detection of livestock needs. Advanced communication between livestock and humans does exist and using it in an applied setting might help decrease stress during handling and better meet the needs of the animals. However, no relevant research on this topic has been conducted yet.

## Ethical Consideration

The question about how we *should* treat farm animals based on their complex social, cognitive, and emotional capacities is a question of philosophy, and more specifically, of animal ethics (198). Several capacity-oriented approaches exist, and these, in one way or another, link the moral status of animals to their abilities (199). However, the role of such abilities and the weight they are assigned will vary based on the different normative frameworks of these theories (200). Two important primary approaches should be separated, as they lead to implications that partly overlap but are also profoundly different in their nature and impact. The moral implications of (farm) animal cognition can first be assessed by *welfare ethics* [understood as an interdisciplinary endeavor among welfare scientists, biologists, veterinarians, and philosophers (201–203)]. Second, one can complete such an assessment by applying *ethical theories that go beyond welfare* [e.g., (204, 205)].

### Welfare Implications

It has been recognized that the links between cognition and welfare are important from an economic perspective in terms of its relation to production success (64). However, from the perspective of animal ethics, it can also be asked whether animal cognition and this connection to animal welfare matters *from the perspective of the animals*.

On the one hand, the nature of animal minds with regard to their capacity to feel pain and other adverse feelings can form the basis for an ethical account of experiential well-being in animals (199). On the other hand, experiential well-being is at the core of one of the most important and most recognized principles in animal ethics, i.e., the *principle of non-maleficence* (206). This principle asks us *not to cause extensive unnecessary harm to others without their consent*, which is a claim that can be specified into several sub-rules. Among them is, most importantly, the rule to *provide for the basic physical and psychological needs* of animals that are under human care (206). This means that welfare ethics establishes an argument that connects physical and psychological needs with welfare and connects welfare with a normative value.

If animal ethics is concerned with animal welfare and welfare is indeed “solely [dependent] on the mental, psychological and cognitive needs of the animals concerned” (207), then the range of connections among capacities, needs and welfare must be considered. For example, learning and memory capacities are assumed to have an impact on the capacity of an animal to cope with housing conditions; thus, these capacities can impact the welfare of the animal (64, 208). Similarly, we might argue that their abilities to recognize and remember conspecifics and to understand the mental states of others (such as their perception and motivations, Table 2) have an impact on the richness and quality of their social life. Such abilities could be important pre-requisites for (or building blocks of) more complex social interactions like empathetically motivated helping behavior or cooperation. The same will be true for animals' general prosocial tendencies and their understanding of fairness. Capacities like these are currently at the center of philosophical, psychological and biological debates, and may even be related to the question whether animals possess the ability to act morally themselves. They will increasingly attract scholarly attention and spur interdisciplinary debates (91, 209–212). Rowlands (212) for example, suggested a de-intellectualized approach to the moral abilities of animals: according to his theory animals can be regarded as moral subjects if their behavior is motivated by moral emotions like empathy.

### Beyond Welfare

However, some ethical problems cannot be fully captured by welfare approaches. If good welfare was the only important ethical premise, then we could potentially instrumentalize, objectify, ridicule, or even kill animals as we like—as long as we did it painlessly. The question is if doing so still constitutes kinds of harms that occur even if the animals do not immediately suffer. In humans, at least, we clearly assume that objectification for example does damage to a human's dignity even if the person herself may not perceive it that way. Therefore, many ethicists meanwhile employ concepts such as respect and dignity in animal ethics as well (213), and develop approaches based on considering the animals' capabilities (205), integrity (214), or rights (215), Such accounts bear the potential to argue beyond the claim of welfare.

In such theories, the complex social and cognitive capacities of animals can play a more direct role in terms of moral qualities. Nussbaum, for example, argued that each species has a set of capabilities which are intrinsically valuable, meaning that behavior based on these capabilities is a value *in itself* and does not just have an instrumental value (205). Carrying out such capabilities is essential to the flourishing of members of that species. Pro-social care behavior falls in this category of capabilities. However, carrying out pro-social care behavior in housing systems that isolate and restrain animals might be impossible. The same might be true if social animals are frequently separated and regrouped according to productivity and reproductive state. In dairy cows, for example, long-term familiarity has an effect on the intensity of social relationships (216). Evidence from other species has suggested that animals have a higher probability of engaging in caring and helping behaviors when they are familiar with the other subject (217). Thus, dairy cows in standard husbandry systems might be restricted to impoverished relationships and social engagement. If the only possible relationships these animals can establish are short-term relationships and if they frequently lose their preferred social partners, this might be considered a welfare issue. However, it could be more than that. If complex capacities in the realm of prosociality, such as caring or helping behaviors, are capabilities that are inherently valuable, then it constitutes a much broader ethical problem that we have established husbandry systems that systematically prevent the animals from developing and maintaining such capacities [for a discussion related to this topic, see (218)].

In contrast to the welfare approach, the animal rights approach asserts that most animals we use as farm animals are *subjects-of-a-life*, i.e., a status for which a range of cognitive, emotional and social capacities are paramount. As such subjects, these animals deserve some basic inviolable rights (215). To build on this idea, biologists and rights philosophers have proposed the claim that animals whose cognitive capacities have high similarities with those of humans should at least be afforded a right to life and freedom and should not be tortured (204). Until now, such claims have been focused on animals that are obviously cognitively complex, e.g., great apes and cetaceans. Future cognition research will increasingly reveal whether the abilities of farm animal species should be interpreted in a substantially different way. The abilities of farm animals might in fact sufficiently resemble the capacities recognized in apes and dolphins and deserve similar moral relevance. Thus, with proceeding research, we can expect more ethical discussions, and some of them will continue to challenge the rather narrow focus of welfare ethics.

## Recommendations for Future Research and Conclusions

Farm animal cognition is a relatively new, but growing, field of research. It provides an excellent opportunity for interdisciplinary work that combines research on animal cognition and animal welfare (48, 208). For instance, paradigms such as the judgement bias test first emerged in human psychological research; now this paradigm is an established test paradigm in applied ethology (219). Similar transfers of other test paradigms are likely to follow and will provide exciting new insights into the minds of farm animals. Indeed, the increased implementation of experimental designs used in human/primate psychological research is highly recommended to improve our understanding about how livestock perceive and interact with their environment.

The attribution (or lack of attribution) of certain cognitive capacities in farm animals is not only relevant for providing adequate welfare but also for consumer choices (220). For example, the tendency to not eat a specific kind of meat increases as more human-like cognitive capacities are attributed to a particular livestock species (221). In contrast, the “dumbing” down of farm animals leads to less moral concern in terms of eating these species (222). Current evidence only scratches the surface of farm animal cognitive capacities, but it already indicates that livestock species possess sophisticated cognitive capacities that are not yet sufficiently acknowledged in welfare legislation. Thus, the recognition of farm animal cognition plays—and will continue to play—a vital role in consumer attitudes as well as in ethical theory.

In this article, we reviewed the evidence on a variety of cognitive traits in farm animals. Certain traits, such as the ability to form categories or the differentiation among individuals, have been thoroughly investigated. However, we identified a lack of research on a diverse set of physico-cognitive capacities (such as numerosity discrimination and object permanence). This knowledge in particular is of key interest to better understand how farm animals perceive their physical environment; this information will improve our design of husbandry environments and enhance the development of management practices.

Finally, we want to emphasize that especially for research on farm animals it is important to know what they are not capable of; this helps us to avoid exposing these animals to stressful situations (83). For instance, the degree to which subjects are able to mentally travel in time is highly relevant to how they anticipate positive or negative future events (208). The “file drawer effect,” i.e., negative findings remain unpublished because they are not novel or exciting enough (223), is thus likely to massively hamper progress on how to adequately address welfare issues.

## Author Contributions

All authors listed have made a substantial, direct and intellectual contribution to the work, and approved it for publication.

### Conflict of Interest Statement

The authors declare that the research was conducted in the absence of any commercial or financial relationships that could be construed as a potential conflict of interest.
